# A global checklist of the parasites of the harbor porpoise *Phocoena phocoena*, a critically-endangered species, including new findings from the Baltic Sea

**DOI:** 10.1016/j.ijppaw.2021.07.002

**Published:** 2021-07-13

**Authors:** Joanna Dzido, Leszek Rolbiecki, Joanna N. Izdebska, Jerzy Rokicki, Tytus Kuczkowski, Iwona Pawliczka

**Affiliations:** a Department of Invertebrate Zoology and Parasitology, Faculty of Biology, University of Gdańsk, Wita Stwosza 59, 80-308, Gdańsk, Poland; b Institute of Oceanography, Faculty of Oceanography and Geography, University of Gdańsk, Morska 2, 84-150, Hel, Poland

**Keywords:** Harbor porpoise, Parasites, Biodiversity, Host-parasite interaction, Odontoceti, Marine mammals

## Abstract

The common harbor porpoise is a widely-distributed marine mammal with three known subspecies, including *P. phocoena phocoena*, with a clearly distinct and critically endangered (CR) subpopulation from the Baltic Sea (Baltic Proper). As part of an assessment of the condition and health threats of these mammals, it is important to conduct parasitological monitoring. The aim of the study was therefore to compare the data on porpoise parasitofauna from this subpopulation with those on porpoises from other areas. The study included 37 individuals from 1995 to 2019; eight species of parasites were found (prevalence 83.8%, mean intensity 724.2, range 2–3940), with a predominance of lung nematodes – *Stenurus minor* (94.7%), *Torynurus convolutus* (69.4%), *Pseudalius inflexus* (63.8%), *Halocercus invaginatus* (22.2%); the highest intensity was recorded for *S. minor* (989, 53–2928). Two species of Anisakidae (*Anisakis simplex* – 33.3%, *Contracaecum* sp. – 20.0%) were found in the digestive tracts, which were a new record for this population. The fluke *Campula oblonga* was found in the livers of 31.3% of porpoises. The tapeworm *Diphylobothrium stemmacephalum* was also recorded in the intestine of one individual; this is typical for these hosts, but previously undetected in the Baltic subpopulation. Parasites coexisted in numerous hosts, constituting a heavy burden for them. The obtained data were compared with those from the *P. phocoena* parasitofauna from other regions, based on a compiled checklist (1809–2021) including all species of porpoise parasites (55 taxa). Compared to the worldwide porpoise parasitofauna checklist, the number of parasites found in the nominative subspecies (Baltic Proper subpopulation) is small: including only 10 taxa (eight in the current study). These species are typical of porpoises and usually the most common; however, the level of infection of Baltic porpoises (intensity and total parasite load) is very high, which can undoubtedly have a negative impact on their condition and overall health.

## Highlights

• The common harbor porpoise is a widely-distributed marine mammal.
• The harbor porpoise population of the Baltic Sea is classified as Critically Endangered (CR).
• The worldwide porpoise parasitofauna checklist the list consists of 55 taxa, including 36 identified to the species rank.
• In the small size of the Baltic subpopulation, only 10 species of parasites were found, but with very high burdens of infection.
• Parasites showing a high level (intensity) of infection, especially lung nematodes, can pose a significant threat to the condition, health and survival of porpoises.

## Graphical abstract

**Figure:**
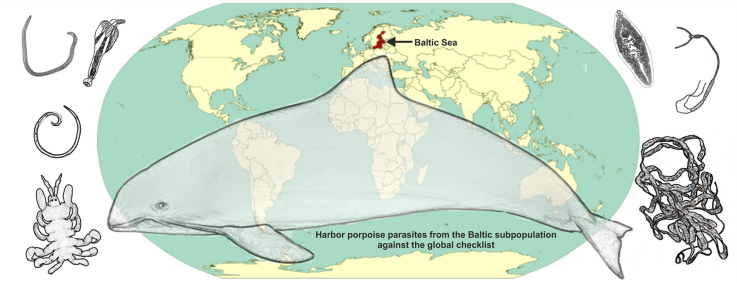

## 1 Introduction

Some of the smallest mammals found in oceanic waters are the porpoises (Cetacea: Odontoceti: Phocoenidae). This group comprises three genera and seven species, four of which occupy the widely-distributed genus *Phocoena* (Committee on Taxonomy, 2020)*.* One of the best known taxa is the harbor porpoise *Phocoena phocoena* (Linnaeus, 1758). Its taxonomy seems to play a significant role in the diversity of its parasitofauna and its importance. The development of parasitofauna follows an evolutionary path associated with the formation of local populations and the scope and possibility of the exchange of host individuals, as well as local behavior patterns, including the quality and diversity of diet.

The harbor porpoise is viewed as a polytypic species, with geographically-varied populations forming three subspecies: *P. phocoena phocoena* (Linnaeus, 1758) the Atlantic harbor porpoise, *P. p. relicta* Abel, 1905 the Black Sea harbor porpoise and *P. p. vomerina* (Gill, 1865) the Pacific harbor porpoise. However, recently it was considered expedient to isolate a fourth subspecies, *P. p. meridionalis* Fontaine et al. (2014)*,* from the southern waters of the Northeast Atlantic off the coasts of Iberia and Mauritania (Fontaine, 2016, Fontaine et al., 2014). All are included in the global The IUCN Red List as being threatened to varying degrees, and the nominative subspecies has been assigned with LC (Least Concern), although there are regional differences here. Therefore the Western Baltic subpopulation has a status Vulnerable (VU), and the most threatened is the Baltic Sea subpopulation, which has been classified as Critically Endangered (CR) (HELCOM, 2013).

The harbor porpoise population of the Baltic Sea drastically decreased in the 20th century: according to data from 1995, there were only 599 individuals (Hiby and Lovel, 1996 as cited in Teilmann, 2011), and this number had fallen to 93 in 2002 (Berggren et al., 2004 as cited in Teilmann, 2011). However, these numbers were estimated based on a small amount of data. A more reliable determination of their numbers was made possible by the data collected as part of Static Acoustic Monitoring of the Baltic Sea Harbor Porpoise (SAMBAH), which in 2014 were estimated at 447 individuals (Pawliczka, 2011; Pawliczka personal comm.). Since the 1990s, regular observations of the harbor porpoise distribution have been conducted in the Polish zone of the Baltic Sea; although their constant presence has been noted, they remain rare animals, with no signs of improvement in their numbers (Pawliczka, 2011).

Genetic studies of *P. p. phocoena* show that a geographic stratification exists, resulting in the formation of two or three subpopulations depending on the source (Wiemann et al., 2010): apart from the mentioned Baltic Sea subpopulation (Baltic Proper), other subpopulations have been identified in the North Sea (including Skattegat) and the Belt Sea (Wiemann et al., 2011). These subpopulations demonstrate a minor dispersal level, amounting to about 1%, as the exchange of specimens between them is low in relation to the total population size (Wiemann et al., 2011). However, these subpopulations are characterized by substantial asymmetry in size, with only several hundred specimens recorded within the Baltic Proper against tens of thousands in the remaining regions (Hammond et al., 2002). Indeed, the harbor porpoises of the Baltic Proper subpopulation differ from other subpopulations of this subspecies in terms of morphology and genetics, and as such, they should be subject to a special level of conservation (Wiemann et al., 2010).

The formation of a local population is probably due to porpoises' strong fidelity to their natal site. Although it was observed that they moved along the coasts, they are usually relatively sedentary and usually do not leave a certain area for a long time (Bjørge and Tolley, 2018). In turn, local conditions also determine the diet, as porpoises are considered not very picky and use the food base available in a given place and season, mainly small fish (Pawliczka, 2011; Winkler et al., 2011). However, their diet in the Baltic Sea differs from that in other regions. The food contains a relatively large proportion of gobies (especially in young porpoises), as well as herring *Clupea harengus* Linnaeus, 1758, Atlantic cod *Gadus morhua* Linnaeus, 1758, and eelpout *Zoarces viviparus* (Linnaeus, 1758). While in the transitional region between the Baltic Sea and the North Sea, the share of herring and gadids is significant, with a much smaller proportion of gobies (Winkler et al., 2011).

Undoubtedly, the existence of local populations may favor the formation of parasitofauna groupings with specific traits. On the other hand, their migration potential, as well as their certain flexibility in terms of food choice, i.e. small fish of various species, may result in the formation of universal parasitofauna patterns for the species throughout its distribution. The aim of the present analysis is to compare data on the parasitofauna of the critically-endangered Baltic Sea subpopulation with that obtained from other subspecies of harbor porpoise with different areas of distribution. The findings may prove valuable in the assessment of the parasitological threats to these rare mammals. Furthermore, an accurate determination of the prevalence may assist the assessment of the condition and health of the hosts.

## 2 Materials and methods

### 2.1 Detection of parasites in *Phocoena phocoena* from the polish coast of the Baltic Sea

The harbor porpoises used in the study were collected in the years 1995–2019; all were found dead on the shore or collected from fishing bycatches on the Polish coast of the Baltic Sea (Baltic Proper, South Baltic). The harbor porpoises were transported to the Hel Marine Station, University of Gdańsk (Hel, Poland). The specimens were stored at −20 °C until further analyses.

Thirty-seven harbor porpoises were examined (Table 1). However, it was not always possible to analyze whole mammals and all their organs. Only the digestive tracts, hearts, lungs and tracheae were available for porpoises no. 42–53, the heads, lungs, tracheae and hearts for porpoises no. 75–84, and only the stomach for porpoise no. 109. Furthermore, data concerning *Stenurus minor* nematodes from porpoises no. 54–60 were not included because they had already been published by Kijewska et al. (2003).

**Table 1:** Sampling details for the harbor porpoises examined with numbers of recovered parasites.

Host catalog no.	Collection date	Locality	Sex (age)	Length [cm]/ weight [kg]	Parasite numbers							
					*C. o.*	*D. s.*	*A. s.*	*C.* sp.	*H. i.*	*P. i.*	*S. m.*	*T. c.*
42^a^	10.1995	Władysławowo	F (4)	165/57			98					7
43^a^	12.1995	Jastarnia	F+ (6)	167/68								47
44^a^	03.1996	Ustka	F (2)	130/35				5				
45^a^	03.1996	Ustka	M (0+)	12738								2
46^a^	03.1996	Jastarnia	M (4)	153/44								
47^a^	03.1996	Rowy	M (2)	135/36								49
48^a^	03.1996	Krynica Morska	F (1)	132/35								
49^a^	04.1996	Rewa-Jastarnia	M (5)	146/45						4		48
50^a^	04.1996	Jarosławiec	M (3)	151/48			3	5				
51^a^	04.1996	Gąski	M (1+)	143/37								
53^a^	07.1996	Jastarnia	M	130/26					15	76		15
54^b^	07.1996	Unieście	M	120/25						12		9
56^b^	09.1997	Władysławowo	M	110/25								
57^b^	12.1997	Jantar (Vistula Spit)	F	117/21			53		13	57		178
58^b^	01.1998	Gulf of Gdańsk	F	114/30			6					1
59^b^	01.1998	Gulf of Gdańsk	F	155/28	41		3			32		
60^b^	11.1998	Ustka	M	134/33	20		11			58		24
61	11.1999	Niechorza	M (1)	120/30					30	89	1668	226
62	12.1999	Puck Bay	M (2)	149/40	4		2	3		17	569	303
63	03.2000	Krynica Morska	M (2)	144/46	12		1	4		71	641	188
64	03.2000	Górki Wschodnie	F (1)	115/29	13					156	834	62
67	08.2008	Ustka	F (1)	131/44	18				2	50	1798	24
68	11.2000	Ustka	F (9)	171/80	162		777	9		64	2928	
69	11.2000	Kuźnica	M (1)	149/44	55		39	2	12	121	1953	
70	01.2001	Dziwnów	M (2)	142/43	5		2			61	566	3
71	03.2000	Jastarnia	M (1)	139/38					35	25	837	31
75^c^	04.2003	Darłówko	F (2)	143/43						34	902	20
76^c^	01.2003	Unieście	M (2)	134/47						16	1200	29
77^c^	02.2003	Darłówko	F (1)	105/33							53	
78^c^	03.2003	Świbno	M (1,5)	119/35						26	1262	41
79^c^	04.2003	Puck Bay	M (2)	137/40						14	800	2
80^c^	04.2003	Puck Bay	M (2)	140/43						56	251	30
84^c^	11.2004	Puck Bay	F (2)	139/36						21	250	8
109^d^	08.2013	Pogorzelica	M	141/32								
136	03.2018	Rowy	M (juv)	127/40						52	756	701
149	07.2019	Dąbki	M (juv)	122		2					534	5
150	07.2019	Ustka	M	82/6								

*A. s.*: *Anisakis simplex*; *C. o.*: *Campula oblonga*; *C.* sp.: *Contracaecum* sp.; *D. s*.: *Diphyllobothrium stemmacephalum*; F: female; F+: pregnant female; *H. i.*: *Halocercus invaginatus*; juv: juvenile; M: male; *P. i.*: *Pseudalius inflexus*; *S. m.*: *Stenurus minor*; *T. c.*: *Torynurus convolutus*.

a The digestive tracts (stomachs, intestines, pancreas, livers and bile ducts), hearts and respiratory tracts (trachea and lungs) were examined.

b Data on parasites *Stenurus minor* in Kijewska et al. (2003).

c The heads, hearts and respiratory tracts (trachea and lungs) were examined.

d The stomach was examined.

The animals were measured to an accuracy of 1 cm and weight to an accuracy of 1 kg, and the sex was determined, followed by a comprehensive parasitological (helminthological) examination. The ear canals, nasal cavity, throat, larynx, trachea, bronchi, lungs, oesophagus, stomach, small intestine, large intestine, pancreas, liver and bile ducts, heart, pulmonary arteries, spleen and kidneys were examined. The liver, kidneys, pancreas and spleen were dissected into smaller pieces and reviewed using a stereoscopic microscope. The trachea and bronchi were cut longitudinally; the lungs were cut along the bronchi, so as to avoid damaging the possible contents, followed by macroscopic examination, and the contents were rinsed with tap water. Similarly, the heart and blood vessels were cut, rinsed with water and the content was observed under a stereoscopic microscope. The contents of the digestive tracts were examined by decantation, in which heavier elements, including parasites, settle faster in water. After allowing the precipitate to settle (20–30 min), the supernatant was carefully poured off and more water was added to the remaining portion. This procedure was repeated one more time, and the parasites were collected from the sediment.

The collected parasites were fixed in 70% ethyl alcohol. The nematodes were cleared in lactophenol to allow identification; some nematodes were mounted in glycerol gelatin or in polyvinyl-lactophenol. Any trematodes or cestodes were stained with alcohol-borax carmine solution and lactic acid carmine, respectively, and then dehydrated in an alcohol series (80, 90, 2 × 99%), cleared in xylene/benzyl alcohol and mounted in Canada balsam (Rolbiecki, 2002, 2007; Rolbiecki et al., 2021).

The prevalence and intensity (range, mean) were calculated to determine the level of host infection (Margolis et al., 1982).

### 2.2 The checklist structure

The checklist was drawn up based on publications (112 items) from the period between 1809 and 2021. The bibliographic search was supplemented by information from Google Scholar, Marine Mammals Research and Conservation Discussion (MARMAM), PubMed, ResearchGate, Scopus, ScienceDirect, Web of Sciences, and World Register of Marine Species (WoRMS). It also contains own unpublished data, marked in the list as “this study”. The species have been arranged in systematic order, and then in alphabetical order. The list further includes information on the microhabitat and geographic distribution of the parasites. Data concerning individual subpopulations of the nominative subspecies are listed (Table 2).

**Table 2:** Parasites species of the harbor porpoises in the Baltic Sea area, based on new records and the literature (for references see the section checklist in this paper).

Parasite	Baltic Proper	Belt Sea	North Sea/Skattegat
APICOMPLEXA			
*Toxoplasma gondii*		+	+
TREMATODA			
*Braunina cordiformis*			+
*Campula oblonga*	+^a^	+	+
*Pholeter gastrosphilus*		+	+
CESTODA			
*Diphyllobothrium stemmacephalum*	+^a^	+	+
*Diphyllobothrium* sp.		+	+
NEMATODA			
*Anisakis simplex*	+^a^	+	+
*Anisakis* sp.			+
*Contracaecum osculatum*		+	
*Contracaecum* sp.	+^a^		
Ascarids		b	b
*Halocercus invaginatus*	+^a^	+	+
*Halocercus taurica*			+
*Halocercus* sp.			+
*Hysterothylacium aduncum*		+	+
*Pseudalius inflexus*	+^a^	+	+
*Stenurus minor*	+^a^	+	+
*Torynurus convolutus*	+^a^	+	+
ACANTHOCEPHALA			
*Bolbosoma* sp.		c	c
*Corynosoma semerme*	+		
*Corynosoma strumosum*	+		
AMPHIPODA			
*Isocymaus delphinii*			+

a This study.

b Andersen (1974) only gives “Danish waters”.

c Not detailed (Herreras et al., 1997).

## 3 Results

### 3.1 Parasites in *Phocoena phocoena* from the polish coast of the Baltic Sea

The studied harbor porpoises were found to contain eight parasite species, classified into digeneans, cestodes and nematodes (Table 1). The overall prevalence, i.e. including all parasites, among the hosts was 83.7%, mean intensity 724.2 and intensity 2–3940.

The predominant parasites were nematodes, particularly the species found in the respiratory system, and sometimes in the heart: *Halocercus invaginatus*, *Pseudalius inflexus*, *Stenurus minor* and *Torynurus convolutus* (Table 3). Of these, *S. minor* was predominant (94.7% of the infected harbor porpoises), followed by *T. convolutus* (69.4%) and *P. inflexus* (63.8%). In addition, the highest infection intensity was found for *S. minor* (range 53–2928; mean 989.0). Furthermore, the infection intensity of *S. minor* in individual specimens (data concerning only harbor porpoises no. 61–64, 67–71, 75–80, 84, 136 and 149) typically reached very high values of several hundred nematodes per host: maximum intensities were 1401 (left ear, porpoise no. 68) and 1527 (right ear, porpoise no. 68) (Table 4).

**Table 3:** Prevalence, intensity and infection site of parasites species collected from the harbor porpoises in the Baltic Proper examined in present study (1995–2019).

Parasite species	Examined host^a^	Prevalence [%]	Intensity range	Mean intensity	Microhabitat
TREMATODA					
*Campula oblonga*	29	31.3	4–162	36.7	liver, bile ducts
CESTODA					
*Diphyllobothrium stemmacephalum*	29	3.4	2	2.0	intestine
NEMATODA					
*Anisakis simplex*	30	33.3	1–777	90.5	stomach, intestine
*Contracaecum* sp.	30	20.0	2–9	4.7	stomach, intestine
NEMATODA					
*Halocercus invaginatus*	36	22.2	2–35	17.8	lungs,
*Pseudalius inflexus*	36	63.8	4–156	49.3	lungs, heart
*Stenurus minor*	19	94.7	53–2928	989.0	middle ear, Eustachian tube, inner ear, nasal cavity, throat, larynx, lungs
*Torynurus convolutus*	36	69.4	1–303	82.1	lungs, heart, trachea

a Selected organs were examined (see Materials and methods).

**Table 4:** Distribution and number of *Stenurus minor* nematodes collected from the harbor porpoises in the Baltic Proper examined in present study (1995–2019).

Host catalog no.	Parasites number						
	Total no.	Left ear	Right ear	Lungs	Throat	Larynx	Nasal cavity
61	1668	685	983				
62	569	569					
63	641	325	316				
64	834	550	284				
67	1798	1148	643	7			
68	2928	1401	1527				
69	1953	841	1112				
70	566	152	414				
71	837	530	307				
75	902	401	501				
76	1200	331	869				
77	53	23	30				
78	1262	314	864		38	46	
79	800	195	605				
80	251	240	11				
84	250	148	98			4	
149	534	322	200				12
136	756	359	397				

In addition, two Anisakidae species were recorded in the gastrointestinal tract of the examined harbor porpoises: the larvae and adults of *Anisakis simplex* in 33.3% of tested harbor porpoises and *Contracaecum* spp. in 20.0% of porpoises. The majority of gastrointestinal nematodes were found in the stomachs (*A. simplex* – 992 ind. *Contracaecum* spp. – 18 ind.), with only individual specimens being present in the intestines (*A. simplex* – 3 ind. *Contracaecum* spp. – 10 ind.). In two harbor porpoises (no. 68 and 69), the stomachs had ulcerative lesions of the gastric mucosa. In addition, the *Campula oblonga* trematode was found in the livers of 31.3% of the harbor porpoises, and two specimens of *Diphylobothrium stemmacephalum* cestode were found in the intestine of a single individual. In addition, the presence of *C. oblonga* was noted in the bile ducts, resulting in their periductular fibrosis.

**Fig. 1:**
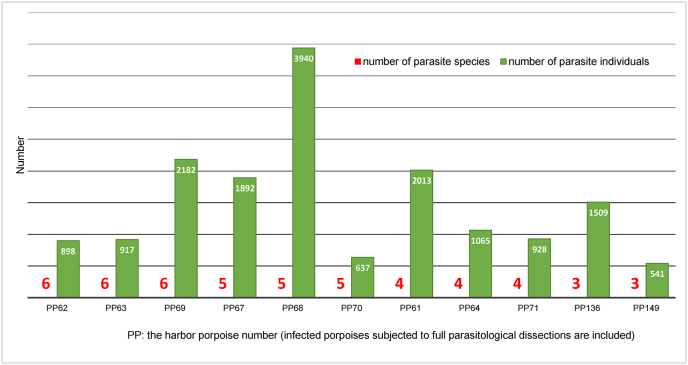
The harbor porpoise parasites load (number of species/number of individuals).

Parasite co-occurrence was also analyzed for 11 harbor porpoises which underwent full dissections, i.e. covering all organs/systems. All specimens contained *S. minor* nematodes, and they were always accompanied by other parasites of the respiratory system, heart or the digestive system (liver, intestines, stomach). Six parasite species were observed in three harbor porpoises, five species in three porpoises, four species in three porpoises and three species in two porpoises (Fig. 1). Regarding the co-occurrence of respiratory tract nematodes of the family Pseudaliidae, three porpoises were found to have four species (*S. minor*, *H. invaginatus, P. inflexus* and *T. convolutus*), five porpoises – three species (*S. minor, P. inflexus, T. convolutus*), one porpoise – three species (*S. minor, H. invaginatus, P. inflexus*), two porpoises – two species each (first – *S. minor* and *P. inflexus* and second – *S. minor* and *T. convolutus*). Porpoise no. 68 (the largest individual studied and probably one of the oldest) was an interesting case, as it had the highest number of *S. minor* but it was also infected with *P. inflexus, C. oblonga*, *A. simplex* and *Contracaecum* spp. A total of 3940 parasite specimens were found in this porpoise.

### 3.2 Biodiversity and geographic distribution of parasites in *Phocoena phocoena*

Fifty-five parasite taxa (including 36 species, 17 identified to the genus level, one to higher taxa, and one as ascarids) have been identified among harbor porpoises from different regions of the world. Of these there was one species of the Protozoa/Metamonada, five taxa of Chromista/Apicomplexa (two identified as species, two to genus level, and one to the subclass), nine Digenea (eight identified as species and one to genus level), 10 Cestoda (six identified as species and four to genus level), 21 Nematoda (12 identified as species, eight to genus level, one as ascarids), six Acanthocephala (four identified as species and two to genus level), two species of Amphipoda, and one species of Copepoda (see below paragraph 3.3.). The highest number of parasite taxa were determined in the nominative subspecies *P. p. phocoena* (42 taxa), followed by *P. p. vomerina* (29), and then *P. p. relicta* (9). Regarding *P. p. phocoena*, the lowest number of taxa were determined in the Baltic Proper subpopulation (10).

### 3.3 A checklist of parasites reported from *Phocoena phocoena*

#### 3.3.1 Metamonada

***Giardia intestinalis*** ( = *G. duodenalis*) (Lambl, 1859)
Microhabitat: large intestinal content^1,2^
Locality: NW Atlantic (Cape Cod^1^), NE Atlantic (Spain^2^)
References: Lasek-Nesselquist et al., 2008^1^; Reboredo-Fernández et al., 2015^2^

#### 3.3.2 Apicomplexa

***Cryptosporidium*** **spp.**
Microhabitat: large intestinal content
Locality: NE Atlantic (Spain)
References: Reboredo-Fernández et al., 2015
***Sarcocystis neurona*** Dubey, Davis, Speer, Bowman, De. Lahunta, Granstrom, Topper, Hamir and Suter 1991
Microhabitat: brain^1-3^
Locality: NE Pacific (British Columbia/Washington^1,3^, California^2^)
References: Barbosa et al., 2015^1^; Rejmanek et al., 2010^2^; Gibson et al., 2011^3^
***Sarcocystis* sp.**
Microhabitat: skeletal musculature^1,2^, tongue^2^
Locality: Davis Strait (Greenland^1,2^)
References: Lehnert et al., 2014^1^; Wunschmann et al., 2001^2^
***Toxoplasma gondii*** (Nicolle et Manceaux, 1908)
Microhabitat: blood antibodies^2,3^, brain^1,5,6^
Locality: NE Atlantic (England and Wales^3^), North Sea (Netherlands^6^), Baltic Sea (Denmark^5^), Mediterranean Sea (Spain^2^), NE Pacific (British Columbia/Washington^1,4^)
References: Barbosa et al., 2015^1^; Cabezón et al., 2004^2^; Forman et al., 2009^3^; Gibson et al., 2011^4^; Herder et al., 2015^5^; Van de Velde et al., 2016^6^
**Coccidia n. det.**
Microhabitat: brain
Locality: NE Pacific (British Columbia/Washington)
References: Barbosa et al., 2015.

#### 3.3.3 Digenea

***Braunina cordiformis*** Wolf, 1903
Microhabitat: stomach^1^, stomach wall^2^
Locality: North Sea (Netherlands^2^), not mentioned^1^
References: Gaskin et al., 1974^1^; Kastelein and Lavaleije, 1992^2^
***Campula oblonga*** Cobbold, 1858
Microhabitat: bile/hepatic ducts^1-3,5-9,13-16,19,24,26,34^, egg in feces^18^, liver^5,6,10,14-17,20-23,25,^^27^^-^^30,32,34^, mammary gland^27^, pancreas^15,23,27,28,30,32^, pancreatic ducts^5,9^, stomach^4,20^, not mentioned^12,31,33^
Locality: NW Atlantic (Newfoundland and Labrador ^4^, New England^13^, Newfoundland^4,31^, Quebec and Maritime Provinces Canada^10^), Davies Strait (Greenland^20,23^), NE Atlantic (British waters^2^, England^14,15^, Wales^15^, Faroe Islands^21^, Firth of Forth^8^, France^3^, Iceland^28^), English Channel/North Sea (Belgium and France^16,17^), North Sea (Belgium^5^, Denmark^7^, Germany^29,32^, Netherlands^17,18,19^, Scotland^14^), North Sea/Baltic Sea (Denmark^1,7^, German^5,22,27^), Baltic Sea^11^ (Denmark^7,30^, Germany^30,32^, Poland^26,30,34^), Norwegian waters^22,28^, NE Pacific (British Columbia^10^, Canada^24^, Friday Harbor Washington^6^, Oregon^9^, Salish Sea^25,33^), not mentioned^11,12^
References: Andersen, 1974^1^; Baker and Martin, 1992^2^; Balbuena et al., 1987^3^; Brattey and Stenson, 1995^4^; Brosens et al., 1996^5^; Ching and Robinson, 1959^6^; Clausen and Andersen, 1988^7^; Cobbold, 1858^8^; Dailey and Stroud, 1978^9^; Fenton et al., 2017^10^; Fernandez et al., 1998^11^; Fraija-Fernández et al., 2015^12^; Geraci, 1978^13^; Gibson and Harris, 1979^14^; Gibson et al., 1998^15^; Jauniaux et al., 2002^16^, 2008^17^; Kastelein et al., 1990^18^; Kastelein and Lavaleije, 1992^19^; Kinze, 1989^20^; Larsen, 1995^21^; Lehnert et al., 2005^22^; 2014^23^; Margolis and Arai, 1989^24^; Norman et al., 2004^25^; Rokicki et al., 1997^26^; Siebert et al., 2001^27^, 2006^28^, 2010^29^, 2020^30^; Smith and Threlfall, 1973^31^; Wunschmann et al., 2001^32^; Zier and Gaydos, 2015^33^, this study^34^
***Campula*** **spp.**
Microhabitat: bile ducts^1,2^
Locality: NW Atlantic (USA^1^), NE Pacific (Salish Sea^2^)
References: Jaber et al., 2013^1^, Norman et al., 2004^2^
***Distomum pallassi*** Poirier, 1885
Microhabitat: not mentioned
Locality: not mentioned
References: Poirier, 1885.
Remarks: accidental record for *P. phocoena* (Price 1932)
***Distomum philocholum*** Creplin, 1845
Microhabitat: liver (bile ducts)
Locality: not mentioned
References: Creplin, 1845.
Remarks: doubtful record for *P. phocoena* (Delyamure, 1957; Price, 1932)
***Opisthorchis tenuicolli*s** (Rudolphi, 1819)
Microhabitat: liver (bile ducts)
Locality: not mentioned
References: Price, 1932.
Remarks: information about this species in *P. phocena* only from this review work
***Pholeter gastrophilus* (**Kossack, 1910**)**
Microhabitat: stomach^3,6,8,9,13,14^, stomach wall^1,2,4,5,7,10,11,12^
Locality: NE Atlantic^3^ (Britain^1^, England^2,4^^,5^, Europe^12^, Ireland^3^, Wales^5^), English Channel/North Sea (France and Belgium^7,8^), North Sea (Belgium and German^3^, Netherlands^8^), North Sea/Baltic Sea (Denmark^6^, Germany^13^), German waters^10^, Norwegian waters^14^, Baltic Sea (Denmark and German^15^), Black Sea (Crimea^9^), not mentioned^11^
References: Aznar et al., 2006^1^; Baker and Martin, 1992^2^; Fraija-Fernandez et al., 2017^3^; Gibson and Harris, 1979^4^; Gibson et al., 1998^5^; Herreras et al., 1997^6^; Jauniaux et al.,; 2002^7^, 2008^8^; Krivokhizhin and Birkun, 1994^9^; Lehnert et al., 2005^10^; Odhner, 1914^11^; Price 1932^12^; Siebert et al., 2001^13^, 2006^14^, 2020^15^
***Synthesium mironovi*** (Krotov et Delyamure, 1952) (=*Orthosplanchnus mironovi*, =*Hadwenius mironovi*)
Microhabitat: duodenum^1^, liver and pancreas^3^, stomach^1,2^, not mentioned^4^
Locality: NE Pacific (Canada^2^, Oregon^1^, Salish Sea^4^), Davis Strait (Greenland^3^)
References: Dailey and Stroud, 1978^1^; Margolis and Arai, 1989^2^; Wunschmann et al., 2001^3^; Zier and Gaydos, 2015^4^
***Synthesium nipponicum*** (Yamaguti, 1951) (=*Hadwenius nipponiucus*)
Microhabitat: duodenum^2^, stomach^1-3^, not mentioned^4^
Locality: NE Pacific (Canada^3^, Friday Harbor Washington^1^, Oregon^2^, Salish Sea^4^)
References: Ching and Robinson, 1959^1^; Dailey and Stroud, 1978^2^; Margolis and Arai, 1989^3^; Zier and Gaydos, 2015^4^

#### 3.3.4 Cestoda

***Bothriocephalus*** **sp.**
Microhabitat: not mentioned
Locality: Danish waters
References: Andersen, 1974.
***Diphyllobothrium lanceolatum*** (Krabbe, 1865)
Microhabitat: not mentioned
Locality: Baltic Sea
References: Schmidt-Ries, 1939 as cited in Delyamure et al., 1985.
***Dibothriocephalus latus*** (Linnaeus, 1758) (=*Diphyllobothrium latum*)
Microhabitat: intestine
Locality: Black Sea
References: Borcea, 1935.
Remarks: doubtful record for *P. phocoena* (Delyamure et al., 1985)
***Diphyllobothrium stemmacephalum*** Cobbold, 1858
Microhabitat: intestine^1,3-13^, not mentioned^2^
Locality: NW Atlantic (Newfoundland and Labrador ^4^), NE Atlantic (England and Wales^9^, Firth of Forth^7^), North Sea (Belgium^5^, Denmark^6^, Netherland^1^), North Sea/Baltic Sea (Denmark^1^, German^5,11^), Baltic Sea (Poland^12^), Black Sea^2,8^ (Crimea^10^), not mentioned^2^
References: Andersen, 1987^1^; Bear, 1932^2^; Borcea, 1935^3^; Brattey and Stenson, 1995^4^; Brosens et al., 1996^5^; Clausen and Andersen, 1988^6^; Cobbold, 1858^7^, Delyamure, 1955^8^; Gibson et al., 1998^9^; Krivokhizhin and Birkun, 1994^10^; Siebert et al., 2001^11^, this study^12^
***Diphyllobothrium*** **sp.**
Microhabitat: intestine^2-5^, stomach^1,5^, not detailed^6^
Locality: NE Pacific (British Columbia^2^), NW Atlantic (Newfoundland and Labrador^1^, Quebec and Maritime provinces Canada^2^), NE Atlantic (Iceland^5^), English Channel/North Sea (Belgium and France^4^), North Sea/Baltic Sea (Denmark^3^), Baltic Sea (Denmark and Germany^6^)
References: Brattey and Stenson, 1995^1^; Fenton et al., 2017^2^; Herreras et al., 1997^3^; Jauniaux et al., 2002^4^; Siebert et al., 2006^5^, 2020^6^
***Monorygma grimaldii*** (Moniez, 1899)
Microhabitat: peritoneum, abdominal cavity
Locality: Davis Strait (Greenland)
References: Lehnert et al., 2014.
***Phyllobothrium delphini*** (Bosc, 1802)
Microhabitat: subcutaneous blubber
Locality: Davis Strait (Greenland)
References: Lehnert et al., 2014.
***Phyllobothrium*** **sp.**
Microhabitat: blubber^1,2^
Locality: NW Atlantic (Quebec and Maritime provinces Canada^1^), NE Atlantic (Irish waters^2^), NE Pacific (British Columbia^1^)
References: Fenton et al., 2017^1^; Rogan and Berrow, 1996^2^
***Pyramicocephalus phocarum*** (Fabricius, 1780)
Microhabitat: stomach
Locality: NE Pacific (Hooper Bay)
References: Rausch and Hilliard, 1970.
***Tetrabothrius*** **sp.**
Microhabitat: stomach
Locality: NW Atlantic (Newfoundland and Labrador)
References: Brattey and Stenson, 1995.

#### 3.3.5 Nematoda

***Anisakis simplex*** (Rudolphi, 1809) (=*Ascaris simplex*)
Microhabitat: duodenal ampulla^14^, intestine^5,13,28,36^, oesophagus^23,28,27,34^, stomach^1-24,26-29,31-36^, not mentioned^10,25^, not detailed^30^
Locality: NW Atlantic (Newfoundland and Labrador^5^), Davis Strait (Greenland^19,23^), NE Atlantic (Britain^2^, British waters^3^, England and Wales^11,20^, Faroe Islands^21^, France^4^, Galicia Spain^1^, Iceland^28^, Scotland^31,35^), English Channel/North Sea (Belgium and France^8,15,16^), North Sea (Belgium^7^, England^13^, Germany^29,34^, Netherlands^16,17,33^), North Sea/Baltic Sea (Denmark^13,14^,Germany^7,22,27^), Baltic Sea (Denmark^30^, German^30,34^, Poland^36^), Norwegian waters^22,28^, Norwegian Sea^32^, Black Sea^6^, NE Pacific (Canada^24^, Strait of Georgia^25^, Oregon^9^), PW Pacific (Japan^21^), not mentioned^10,26^
References: Abollo et al., 1998^1^; Aznar et al., 2006^2^; Baker and Martin, 1992^3^; Balbuena et al., 1987^4^; Brattey and Stenson, 1995^5^; Borcea, 1935^6^; Brosens et al., 1996^7^; Clausen and Andersen, 1988^8^; Dailey and Stroud, 1978^9^; Davey, 1971^10^; Gibson et al., 1998^11^; Gibson and Harris, 1979^12^; Herreras et al., 1997^13^, 2004^14^; Jauniaux et al., 2002^15^, 2008^16^; Kastelein and Lavaleije, 1992^17^; Kagei et al., 1967 as cited in Smith, 1989^18^; Kinze, 1989^19^; Kirkwood et al., 1997^20^; Larsen, 1995^21^; Lehnert et al., 2005^22^, 2014^23^; Margolis and Arai, 1989^24^; Paggi et al., 1998^25^; Rudolphi, 1809^26^; Siebert et al., 2001^27^, 2006^28^, 2010^29^, 2020^30^; Smith, 1989^31^, Ugland et al., 2004^32^; Van Beurden et al., 2015^33^; Wunschmann et al., 2001^34^; Young, 1972^35^; this study ^36^
Remarks: The presence of *Anisakis simplex* in the Black Sea is questionable (Herreras et al., 1997).
***Anisakis simplex*** **s. s.**
Microhabitat: stomach^2,3^, not mentioned^1^
Locality: NE Atlantic (Spain^3^); PE Pacific (Strait of Georgia^1^); PW Pacific (southern Hokkaido Japan^2^)
References: Katahira et al., 2021^2^; Mattiucci et al., 1997^1^; Pons-Bordas et al., 2020^3^
Remarks: *Anisakis simplex* sensu stricto is one of the three sibling species of *A. simplex* complex (together with *A. pegreffii* Campana-Rouget et Biocca, 1955 and *A. berlandi* Mattiucci, Cipriani, Webb, Paoletti, Marcer, Bellisario, Gibson et Norman et al., 2004 ) (see Mattiucci et al., 2014). Distinguishing between these species requires careful analysis, therefore specimens identified morphologically are often treated as *A. simplex* sensu lato. In the above studies, the presence of *A. simplex* s. s. was confirmed using DNA methods.
***Anisakis typica*** (Diesing, 1860) (=*Ascaris typica*)
Microhabitat: stomach^1,2^
Locality: NE Atlantic (Scotland^1^), not mentioned^2^
References: Stiles and Hassall, 1899^2^; Young and Lowe, 1969^1^
***Anisakis* sp.**
Microhabitat: stomach^1-6^, not mentioned^7^
Locality: NW Atlantic (Bay of Fundy^3^, Quebec and Maritime Provinces Canada^1^), NE Atlantic (Scotland^5,6^); North Sea/Norwegian Sea (Norway^4^); NE Pacific (British Columbia^1^, Salish Sea^2,7^)
References: Fenton et al., 2017^1^; Norman et al., 2004^2^; Scott and Fisher, 1958^3^; Vik, 1964^4^; Young 1972^5^; Young and Lowe, 1969^6^; Zier and Gaydos, 2015^7^
**Ascarids n. det.**
Microhabitat: feces^2^, stomach^1,3^
Locality: North Sea (Netherlands^2^), North Sea/Baltic Sea (Denmark^1^), NE Pacific (Washington^3^)
References: Andersen, 1974^1^; Kastelein et al., 1990^2^; Scheffer and Slipp, 1948^3^
***Contracaecum osculatum* (Rudolphi, 1802)**
Microhabitat: stomach^1,2^, intestine^1,2^, not detailed^3^
Locality: NW Atlantic (Newfoundland and Labrador^1^), NE Atlantic (Iceland^2^), Baltic Sea (Denmark and Germany^3^)
References: Brattey and Stenson, 1995^1^; Siebert et al., 2006^2^, 2020^3^
***Contracaecum*** **sp.**
Microhabitat: intestine^5^,stomach^1,2,4,5^, not mentioned^3^
Locality: NW Atlantic (Bay of Fundy^2^, Newfoundland^3^, Quebec and Maritime provinces Canada^1^), NE Atlantic (Scotland^4^), Baltic Sea (Poland^5^), NE Pacific (British Columbia^1^)
References: Fenton et al., 2017^1^; Scott and Fisher, 1958^2^; Smith and Threlfall, 1973^3^; Smith, 1989^4^, this study^5^
***Crassicauda*** **sp.**
Microhabitat: blubber^1,2,4,8^, cranial sinues^6^, frontal sinuses^3^, mammary^2,10^, muscle^10^, subcutaneous thoracic wall^1^, subcutis^9,10^, perimuscular fascia and subcutaneous fat^7^, not mentioned^11^, not detailed^5^
Locality: NW Atlantic (Quebec and Maritime provinces Canada^4^, Gulf of Saint Lawrence^3^), Davies Strait (Greenlnad^7,10^), NE Atlantic (British waters^1^, England and Wales^5^), Black Sea (Crimea^6^), NE Pacific (British Columbia^4^, Canada^8^, Oregon^2^, Salish Sea^9,11^).
References: Baker and Martin, 1992^1^; Dailey and Stroud, 1978^2^; Faulkner et al., 1998^3^; Fenton et al., 2017^4^; Gibson et al., 1998^5^; Krivokhizhin and Birkun, 1994^6^; Lehnert et al., 2014^7^; Margolis and Arai, 1989^8^; Norman et al., 2004^9^; Wunschmann et al., 2001^10^; Zier and Gaydos, 2015^11^
***Halocercus invaginatus*** (Quekett, 1841) (=*Filaria inflexocaudata*, =*H. inflexocaudatus*, =*H. ponticus*, =*Pseudalius tumidus*, =*Strongylus invaginatus*)
Microhabitat: branchioles^18^, lungs^1,2,4-12,14-16,18,19-30,33,34,36^, pulmonary blood vessels^18^, respiratory tract^17^, trachea^13^, not montioned^3,31,32,35^
Locality: NW Atlantic (Bay of Foundy^3^, Newfoundland^31^), Davis Strait (Greenland^18^), NE Atlantic (British waters^4^, England and Wales^12^, France^11^, Faroe Islands^15^, Galicia Spain^1^, Iceland^28^, Irish waters^23^, Norwegian waters^5,16^), North Sea (Belgium^10^,German^10,17,^ Netherlands^10,13,30^), North Sea/Baltic Sea (Denmark^2^, German^16^), Baltic Sea^26^ (Denmark^29^, German^27,29^, Poland ^19,24,29,33,36^), Marmara Sea (Turkey^21^), Azov Sea/Black Sea^8^ (Crimea^14^, Turkey^34^), NE Pacific (Oregon^7,32^, Salish Sea^35^, San Francisco Bay^9,11^, Monterey Bay^20^, Vancouver Island^3^, Washington^25^), not mentioned^6,22^
References: Abollo et al., 1998^1^; Andersen, 1974^2^; Arnold and Gaskin, 1975^3^; Baker and Martin, 1992^4^; Balbuena et al., 1994^5^; Baylis and Daubney, 1925^6^; Dailey and Stroud, 1978^7^; Delyamure, 1955^8^; Dougherty, 1943^9^; Van Elk et al., 2019^10^; Gibson and Harris, 1979^11^; Gibson et al., 1998^12^; Kastelein and Lavaleije, 1992^13^; Krivokhizhin and Birkun, 1994^14^; Larsen, 1995^15^; Lehnert et al., 2005^16^, 2007^17^, 2014^18^; Łukasiak, 1939^19^; Moser and Rhinehart, 1993^20^; Pekmezci et al., 2013^21^; Quekett, 1844^22^; Rogan and Berrow, 1996^23^; Rokicki et al., 1997^24^; Scheffer and Slipp, 1948^25^; Schmidt-Ries, 1939^26^, Schneider, 1866^27^; Siebert et al., 2006^28^, 2020^29^; Slob et al., 1996^30^; Smith and Threlfall, 1973^31^; Stroud and Roffe, 1979^32^; Szefer et al., 1998^33^; Veryeri, 2012^34^; Zier and Gaydos, 2015^35^, this study^36^
***Halocercus taurica*** Delyamure, 1942
Microhabitat: lungs^2-7^, not mentioned^1^
Locality: NW Atlantic (Bay of Foundy^1^), NE Atlantic (England and Wales^3^, Irish waters^6^), North Sea (Netherlands^7^), Marmara Sea (Turkey^5^), Azov Sea/Black Sea^2^ (Crimea^4^), NE Pacific (Vancouver Island^1^)
References: Arnold and Gaskin, 1975^1^; Delyamure, 1955^2^; Gibson et al., 1998^3^; Krivokhizhin and Birkun, 1994^4^; Pekmezci et al., 2013^5^, Rogan and Berrow, 1996^6^; Slob et al., 1996^7^
***Halocercus*** **sp.**
Microhabitat: lungs^1-3,5^, not mentioned^4,6^
Locality: NW Atlantic (Newfoundland^4^, Greenland^5^, Quebec and Maritime provinces Canada^1^), English Channel/North Sea (Belgium and France^2^), NE Pacific (British Columbia^1^, Salish Sea^3,6^)
References: Fenton et al., 2017^1^; Jauniaux et al. 2002^2^; Norman et al., 2004^3^; Smith and Threlfall, 1973^4^; Wunschmann et al., 2001^5^; Zier and Gaydos, 2015^6^
***Hysterothylacium aduncum*** (Rudolphi, 1802)
Microhabitat: stomach and intestine^1^, not detailed^2^
Locality: North Sea/Baltic Sea (Denmark^2^), Baltic Sea (Denmark/Germany^2^)
References: Herreras et al., 1997^1^; Siebert et al., 2020^2^
***Pharurus* sp.** (=*Pseudostenurus*)
Microhabitat: not mentioned
Locality: NW Atlantic (Newfoundland)
References: Smith and Threlfall, 1973
Remarks: doubtful record for *P. phocoena* (Arnold and Gaskin, 1975)
***Pharurus dalli*** (Yamaguti, 1951) (=*Irukanema dalli*)
Microhabitat: not mentioned
Locality: NW Atlantic (Newfoundland)
References: Smith and Threlfall, 1973
Remarks: doubtful record for *P. phocoena* (Arnold and Gaskin, 1975)
***Phocascaris*** **sp.**
Microhabitat: stomach, intestine
Locality: NW Atlantic (Newfoundland and Labrador)
References: Brattey and Stenson, 1995.
***Pseudalius inflexus*** (Rudolphi, 1808) (=*Prosthecosacter inflexus*, =*Strongylus inflexus*)
Microhabitat: airways^16,17^, blood vessels^17,7^, blowhole^14^, heart^4,5,7,8,15,29, 33,34, 39^, bronchi^1,2,4-10, 14,15,18,26,31,33,34^, egg in feces^19^, inner ear^13^, lower airways^27^, lungs^5,11,12,14,15,18,20-24,28-30,32,34-39^, oesophagus^32^, pulmonary vessels^4,8,10-12,15,16,18,20,26,34,33^, respiratory tract^25^, right ventricle of heart^12,16,20,26,32^, trachea^15,20^, not mentioned^3^
Locality: NW Atlantic (Bay of Fundy^3^, Labrador^14^, Quebec and Maritime provinces Canada^12^), NE Atlantic (British waters^4^, England and Wales^15,18,22^, Faroe Islands^23^, Firth of Forth^10^, France^5,14^, Iceland^34^, Irish waters^28^, Norwegian waters^6,24,34^), English Channel (Luc-sur-Mer France^1^, Marazion England^27^), English Channel/North Sea (Belgium and France^16,17^), North Sea (Belgium^8,11^, England^14^, Germany^25,32,38^, Netherlands^3,11,17,19,20,36^), North Sea/Baltic Sea (Denmark^2,9^, German^8,24,33^), Baltic Sea^30,31^ (Denmark^35,14^, Germany^13,35,38^, Poland^29,35,37,39^), NE Pacific (British Columbia^12^), Davies Strait (Greenland^21^), not mentioned^7,26^
References: Abeloos, 1932^1^; Andersen, 1974^2^, Arnold and Gaskin, 1975^3^; Baker and Martin, 1992^4^; Balbuena et al., 1987^5^, 1994^6^; Baylis and Daubney, 1925^7^; Brosens et al., 1996^8^; Clausen and Andersen, 1988^9^; Cobbold, 1858^10^; Van Elk et al., 2019^11^; Fenton et al., 2017^12^; Gabel et al., 2020^13^; Gibson and Harris, 1979^14^; Gibson et al., 1998^15^; Jauniaux et al., 2002^16^, 2008^17^; Jepson et al., 2000^18^; Kastelein et al., 1990^19^; Kastelein and Lavaleije, 1992^20^; Kinze, 1989^21^; Kirkwood et al., 1997^22^; Larsen, 1995^23^; Lehnert et al., 2005^24^, 2007^25^; Quekett, 1844^26^; Perrett et al., 2004^27^; Rogan and Berrow, 1996^28^; Rokicki et al., 1997^29^; Schmidt-Ries, 1939^30^; Schneider, 1866^31^; Siebert et al., 2010^32^, 2001^33^, 2006^34^, 2020^35^; Slob et al., 1996^36^; Szefer et al., 1998^37^; Wunschmann et al., 2001^38^, this study^39^
***Pseudoterranova decipiens*** (Krabbe, 1878) (=*Porrocaecum decipiens*)
Microhabitat: not mentioned
Locality: NE Pacific (Salish Sea)
References: Zier and Gaydos, 2015.
***Pseudoterranova* sp.** (=*Porrocaecum*)
Microhabitat: stomach
Locality: NW Atlantic (Bay of Fundy)
References: Scott and Fisher, 1958.
***Stenurus minor*** (Kuhn, 1829) (=*Prosthecosacter minor*, =*Pseudalius minor*, =*Stenurus phocoenae*, =*Strongylus minor*)
Microhabitat: auditory sinuses^24,26,15^, blood vessels^5,15^, blowhole^16^_,_ ears^7,9,20,23,36,41^, bronchi^1,5,10,14,16^, cavum tympani^15,29,33^, cranial sinuses^4,12,22,24,27,30,39^, Eustachian tube^19,25,31, 34,35, 37,39,43^, head sinuses^16^, inner ear^19,40,43^, intestine^34^, larynx^31,43^, lungs^11,16,21,24,28,31,32,38,43^, middle ears^3,6,17-19,40,43^, mouth cavity^10^, nosal sinuses^15^, nosal and head cavites^31,43^, nosal passage^10^, oesophagus^10,35^, peribullar cavity^25,34,35,37^, pterygoid sinuses^13^, tympanic bullae^16,26,27,31,39,5^, respiratory tract^35^, sinuses^8,33^, stomach^10,35^, throat^43^, venous sinuses^29^, not mentioned^2,42^, not detailed^9^
Locality: NE Atlantic (British waters^3^, England and Wales^16,21^, Faroe Islands^23^, France^4^, Iceland^35^, Irish waters^30^, Norwegian waters^24^), English Channel/North Sea (Belgium and France^17,18^), North Sea (Belgium^6,11^, Denmark^40^, England^15^, German^26,36,40,41)^, Netherlands^2,11,18,27,35,38^), North Sea/Baltic Sea (Denmark^1,7,27^, German^6,24,27,34^), Baltic Sea^32^ (Denmark^37,40^, Germany^14,37,40,41^, Poland^19,31,37,43^), NW Atlantic (Quebec a Maritime provinces Canada^13^, Gulf of Saint Lawrence^12^, Bay of Fundy^2^, Newfoundland^2^), Davis Strait (Greenland^20,25,41^), Marmara Sea (Turkey^28^), Azov Sea/Black Sea^9^ (Crimea^22^), NE Pacific (Oregon^8,39^, Salish Sea^42^, San Francisco Bay^10^), not mentioned^5,29,33,42^
References: Andersen (1974)^1^; Arnold and Gaskin (1975)^2^; Baker and Martin (1992)^3^; Balbuena et al. (1987)^4^; Baylis and Daubney (1925)^5^; Brosens et al. (1996)^6^; Clausen and Andersen (1988)^7^; Dailey and Stroud (1978)^8^; Delyamure (1957)^9^; Dougherty (1943)^10^; Van Elk et al., 2019^11^; Faulkner et al. (1998)^12^; Fenton et al. (2017)^13^; Gabel et al. (2020)^14^; Gibson and Harris (1979)^15^; Gibson et al. (1998)^16^; Jauniaux et al. (2002)^17^, 2008^18^; Kijewska et al. (2003)^19^; Kinze (1989)^20^; Kirkwood et al. (1997)^21^; Krivokhizhin and Birkun (1994)^22^; Larsen (1995)^23^; Lehnert et al. (2005)^24^, 2014^25^, 2007^26^; Morell et al. (2017)^27^; Pekmezci et al. (2013)^28^; Quekett (1844)^29^; Rogan and Berrow (1996)^30^; Rokicki et al. (1997)^31^; Schmidt-Ries (1939)^32^, Schneider (1866)^33^; Siebert et al. (2001)^34^, 2006^35^, 2010^36^, 2020^37^; Slob et al. (1996)^38^; Stroud and Roffe (1979)^39^; Wohlsein et al. (2019)^40^; Wunschmann et al. (2001)^41^, Zier and Gaydos (2015)^42^, this study^43^
***Stenurus*** **sp.**
Microhabitat: lungs^1^, not mentioned^2^
Locality: NW Atlantic (Quebec and Maritime Provinces Canda^1^), NE Pacific (British Columbia^1^, Salish Sea^2^)
References: Fenton et al. (2017)^1^; Zier and Gaydos (2015)^2^
***Torynurus convolutus*** (Kühn, 1829) (=*Pharurus convolutus*, =*Prosthecosacter convolutus*, =*Pseudalius convolutus*, =*Strongylus convolutus*, = *Torynurus bicostatus*)
Microhabitat: air sacs^30^, airways^15,16^, blood vessels^16,23^, blowhole^11,13^, ear sinuses^11^, bronchi^1,3-7,9,13,14,17,23,28-30,33^, heart^29,36^, larynx^25,30^, lungs^2,8,10,12-14,17,19-21,24-27,30-34,36^, oesophagus^13,14,31^, pharynx^6^, pulmonary blood vessels^7,13,29^, respiratory tract^22^, trachea^13,14,18,25,36^, not mentioned^2,35^
Locality: NE Atlantic^2^ (British waters^3^, England and Wales^13,14,17,20^, Firth of Forth^7^, France^4^, Irish waters^24^, Iceland^30^), Davies Strait (Greendland^19^), Norwegian waters^5,21,30^, English Channel (Luc-sur-Mer France^1^), English Channel/North Sea (Belgium and France^15,16^), North Sea (Belgium^6,10^, England^13^, Germany^10,22,29,31,34^, Netherlands^2,10,16,18,33^), North Sea/Baltic Sea (German^6,21,29^), Baltic Sea^27,28^ (Denmark^13,32^, Germany^29,32,34^, Poland^25,32,36^), NW Atlantic (Bay of Fundy^2^, Gulf of Saint Lawrence^11^, Labrador^13^, New Brunswick^13^, Quebec a Maritime provinces Canada^12^), NE Pacific (British Columbia^12^, Oregon^8^, Salish Sea^35^, Washington^26^, San Francisco Bay^9^, Vancouver Island^2^); not mentioned^23^
References: Abeloos (1932)^1^; Arnold and Gaskin (1975)^2^; Baker and Martin (1992)^3^; Balbuena et al. (1987)^4^,1994^5^; Brosens et al. (1996)^6^; Cobbold (1858)^7^; Dailey and Stroud (1978)^8^; Dougherty (1943)^9^; Van Elk et al., 2019^10^; Faulkner et al. (1998)^11^; Fenton et al. (2017)^12^; Gibson and Harris (1979)^13^; Gibson et al. (1998)^14^; Jauniaux et al. (2002)^15^, 2008^16^; Jepson et al. (2000)^17^; Kastelein and Lavaleije (1992)^18^; Kinze (1989)^19^; Kirkwood et al. (1997)^20^; Lehnert et al. (2005)^21^, 2007^22^; Quekett (1844)^23^; Rogan and Berrow (1996)^24^, Rokicki et al. (1997)^25^; Scheffer and Slipp (1948)^26^; Schmidt-Ries (1939)^27^, Schneider (1866)^28^; Siebert et al. (2001)^29^, 2006^30^, 2010^31^, 2020^32^; Slob et al. (1996)^33^; Wunschmann et al. (2001)^34^; Zier and Gaydos (2015)^35^, this study^36^

#### 3.3.6 Acanthocephala

***Bolbosoma capitatum*** (von Linstow, 1880)
Microhabitat: intestine
Locality: NE Atlantic (England and Wales)
References: Gibson et al. (1998).
***Bolbosoma*** **sp.**
Microhabitat: intestine^1,2^, stomach^3^
Locality: NW Atlantic (Newfoundland and Labrador ^1^), North Sea/Baltic Sea (Denmark^3^), Pacific (Oregon^2^)
References: Brattey and Stenson (1995)^1^; Dailey and Stroud (1978)^2^; Herreras et al. (1997)^3^
***Corynosoma alaskensis*** Golvan, 1959
Microhabitat: intestine
Locality: Bering Sea (Hooper Bay, Alaska)
References: Golvan, 1959.
***Corynosoma semerme*** (Forssell, 1904) (=*Echinorhynchus semermis*)
Microhabitat: intestine^1-3^
Locality: Baltic Sea (Finland^1,2^), not mentioned ^3^
References: Forssell, 1904^1^, 1905^2^; Lühe, 1911^3^
***Corynosoma strumosum*** (Rudolphi, 1802) (=*Echinorhynchus strumosus*)
Microhabitat: intestine^1,2,3,5^, not mentioned^4^
Locality: NE Atlantic (Icelandic^5^), Baltic Sea (Finland^1,2^), NW Pacific (Hokkaido Japan^4^), not mentioned^3^
References: Forssell (1904)^1^, 1905^2^; Lühe (1911)^3^; Sasaki et al. (2019)^4^; Siebert et al. (2006)^5^
***Corynosoma*** **spp.**
Microhabitat: intestine
Locality: NE Pacific (Canada)
References: Margolis and Arai (1989).

#### 3.3.7 Amphipoda

***Isocyamus delphinii*** (Guérin-Méneville, 1836)
Microhabitat: skin^1-3^
Locality: English Channel/North Sea (Belgium and France^1^), North Sea (Germany^2^, Netherlands^3^)
References: Jauniaux et al. (2002)^1^; Lehnert et al. (2007)^2^; Stock, 1973a a,b^3^;
***Isocyamus deltobranchium*** Sedlak-Weinstein, 1992
Microhabitat: skin
Locality: North Sea (Germany, Netherlands)
References: Lehnert et al., 2021.

#### 3.3.8 Copepoda

***Pennella balaenopterae*** Koren et Danielssen, 1877
Microhabitat: skin
Locality: Aegean Sea (Bodrum Peninsula Turkey)
References: Danyer et al. (2014).

## 4 Discussion

The present study examined the parasitofauna of the harbor porpoise *P. p. phocoena* from the Baltic Proper (south) subpopulation based on examinations of 37 specimens collected over a period of 24 years. The findings indicate the regular occurrence of eight helminth species, which have also been recorded in other studies from different regions of the world (see checklist). The results of the survey in the present study included the first finding of *D. stemmacephalum* cestodes and Anisakidae nematodes in the area. However, it should be noted that individual parasites exhibit different relationship ranges with harbor porpoises, reflected in the incidence rate and infection intensity. Typical parasites include the *C. oblonga* trematode, *D. stemmacephalum* cestode, *H. invaginatus*, *P. inflexus*, *S. minor* and *T. convolutes* nematodes; some of which are specific parasite species for this host (Delyamure, 1955; Arnold and Gaskin, 1975; Delyamure et al., 1985).

Undoubtedly, the most commonly observed parasites are the nematodes: their infection prevalence typically reaches very high values, e.g. for *P. inflexus* it ranges from 89.0% (Belgian and German coasts), 88.0% (coast of England and Wales) to 34.4% (Norwegian waters) (Balbuena et al., 1994; Brosens et al., 1996; Gibson et al., 1998). A high prevalence was also noted in the present study (63.8%), and an earlier study of southern Baltic Sea recorded a level of 88.2% (Rokicki et al., 1997). Similarly, the prevalence of *S. minor* was found to be high as 94.7% in the presently-studied southern Baltic Sea population; this value is significantly higher than in previous studies from this region (47.0%). Very high prevalence values were also observed in other regions: 86.0 and 95% (consistency) in Greenland, and 88.0% off the coast of England and Wales (Rokicki et al., 1997; Gibson et al., 1998; Lehnert et al., 2014).

*Torynurus convolutus* has also demonstrated a very high prevalence in the southern Baltic Sea, i.e. 82.3% in previous studies and 69.4% in the present study, with lower levels observed in other regions: 49.0% off the coast of England and Wales, 44.0% off the Belgian and German coasts and 42.2% in Norwegian waters (Balbuena et al., 1994; Brosens et al., 1996; Rokicki et al., 1997; Gibson et al., 1998). Interestingly, regarding *H. invaginatus*, very high prevalence values were observed in Norwegian waters (98.4%), but considerably lower ones in other regions: prevalence was found to be 22.2% (present study) and only 11.8% (previously) in the southern Baltic Sea, and as low as 1.2% off the coast of England and Wales (Balbuena et al., 1994; Brosens et al., 1996; Rokicki et al., 1997; Gibson et al., 1998).

In contrast, the prevalence of *C. oblonga* trematode infection ranged from 42.2%, off the coast of England and Wales, to 28.0%, off the Belgian and German coasts, to 7.5%, around Newfoundland and Labrador (Brattey and Stenson, 1995; Brosens et al., 1996; Gibson et al., 1998). Currently, for the southern Baltic Sea, this value was 31.3%; this value is considerably lower than in the preceding study period, where only 5.9% was recorded (Rokicki et al., 1997).

The *D. stemmacephalum* cestode*,* although it was described from *P. phocoena* and has regularly been found in harbor porpoises, typically exhibits a low prevalence, ranging from 11.0% (Belgian and German coasts), 6.9% (Newfoundland and Labrador), 4.0% (coast of England and Wales), 3.4% (present, southern Baltic Sea), to 2.9% (Danish waters) (Brattey and Stenson, 1995; Brosens et al., 1996; Herreras et al., 1997; Gibson et al., 1998). In the present study on the southern Baltic Sea, it was only found in a single host. This cestode is a typical parasite of different toothed whales, and perhaps the prevalence is linked to the size of the host species reservoir, i.e. only one whale species is constantly present in the Baltic Sea, or the different availability of intermediate hosts.

A high prevalence was also observed for *A. simplex* and *Contracaecum* spp. nematodes; however, regarding the latter, most data concerns *C. osculatum* or specimens without any identification to a species-level, but that are supposed to be of this genus. The particularly high infection parameters of *C. osculatum* in harbor porpoises are related to the widespread occurrence of these nematodes in the Phocidae as other final hosts. For example, *C. osculatum* exhibited 83.8/75.9% (stomach/intestine) prevalence at Newfoundland and Labrador (Brattey and Stenson, 1995).

In turn, the level of harbor porpoise infection with *A. simplex* varied from 60.0% (Greenland; Lehnert et al., 2014), 59.5% (coast of England and Wales; Gibson et al., 1998), 47.5% (Newfoundland and Labrador; Brattey and Stenson, 1995), 38.6% (Danish waters; Herreras et al., 1997), 33.3% (southern Baltic Sea; present), 33.0% (Belgian and German coasts; Brosens et al., 1996).

One important issue concerns the predominance of nematodes inhabiting the respiratory system. Lungworms of the family Pseudaliidae were here represented by *S. minor*, a species typical for harbor porpoise and found in all *P. phocoena* subspecies; However, it exhibited very high infection parameters in the present study (prevalence 94.7%, mean intensity 989.0, intensity 53–2928). The particularly significant value in this case is the infection intensity, which reflects the host parasite load, i.e. its pressure on the host. This high mean intensity in the population stemmed from the very high prevalence observed in certain host specimens; e.g. 2928 specimens of these parasites were found in one porpoise (no. 68), including 1401 in the left ear and 1527 in the right ear. Although opinions differ on the significance of these nematodes for the health and overall condition of harbor porpoises (Delyamure, 1955; Geraci, 1978), such a high prevalence must surely have an influence on the functioning of this system/organ, which is important for this marine mammal. Examinations of harbor porpoise from the Polish Baltic zone have already reported the presence of pathological lesions associated with a similar prevalence of these parasites (83.3%, 779.6 ind. per ear) (Kijewska et al., 2003), suggesting a possible disruption of their echolocation capabilities.

Three other Pseudaliidae species were found to have a lower prevalence in the present study (total prevalence 77.8%, mean intensity 61.0): *Torynurus convolutus* (69.4%, 82.1), *Pseudalius inflexus* (63.8%, 49.3) and *Halocercus invaginatus* (22.2%, 17.8). The same three species were determined i.a. in the study on harbor porpoises from the German Wadden Sea (the southeastern part of the North Sea) in the period 2006–2018 (Reckendorf et al., 2021). It was noted that infection with pulmonary nematodes and associated secondary bronchopneumonia may have a profound impact on the health status of harbor porpoises in this region, and may even constitute the main cause for harbor porpoise mortality in the North Sea (Siebert et al., 2001, 2006; Jauniaux et al., 2002; Lehnert et al., 2005; Van Elk et al., 2019). However, the total prevalence of infection with these nematodes was lower than in the present study, amounting to 45.6%, and the majority of infected harbor porpoises were found to have moderate or acute infection of 38.1% and 39.0%, respectively, of harbor porpoises with a positive test result for the presence of pulmonary nematodes. In contrast, 22.9% of other specimens were found to demonstrate non-severe infection symptoms (Reckendorf et al., 2021). Based on an analysis of data from different areas of the range of *P. p. phocoena*, the authors suggest that despite the higher prevalence of infection in the northern regions (Norway, Iceland). The parasitism, is typically characterized by mild symptoms, whereas cases of severe symptoms are more numerous in research from the North Sea and Baltic Sea.

An analysis of parasite checklists of harbor porpoises according to subspecies and distribution indicates the existence of other potential threats to this cetaceans. Representatives of the Apicomplexa, including *Toxoplasma gondii,* or the genera *Cryptosporidium* and *Sarcocystis* are sporadically mentioned. However, the absence of more comprehensive data probably stems from the fact that these unicellular parasites are rarely included in parasitological analyses of whales, which is usually include directed towards helminths. *Toxoplasma gondii*, whose life cycle is linked with terrestrial environment (final host – cat, intermediate – rodents), is often analyzed in the context of importance for accidental hosts, particularly humans, where it may have a negative impact on fetal development in the form of congenital toxoplasmosis, which can contribute to abortions or malformations. Fortunately, the knowledge base concerning the neurological importance of *Toxoplasma* for different hosts is also increasing, including its contribution to so-called *risky behavior* (Webster, 2001; Conrad et al., 2005). Other records of this parasite in aquatic species, including marine mammals, suggest that its transmission and dispersal have considerably wider potential than that resulting from the simple realization of its life cycle. Although atypical hosts do not enable its sexual reproduction, they may also suffer health consequences associated with contact with the parasite. Both *T. gondii* infection and toxoplasmosis have been described around the world in marine mammals, including whales. Congenital toxoplasmosis related to fetus infection has been reported in the Risso's dolphin *Grampus griseus* (Resendes et al., 2002), and in the Indo-Pacific bottlenose dolphin *Tursiops aduncus* (Jardine and Dubey, 2002). Cases of toxoplasmosis have also been recorded in the beluga whale *Delphinapterus leucas* (Mikaelian et al., 2000), Indo-Pacific humpback dolphin *Sousa chinensis* (Bowater et al., 2003), spinner dolphin *Stenella longirostris* (Migaki et al., 1990), as well as in numerous pinnipeds (Migaki et al., 1977; Holshuh et al., 1985; Conrad et al., 2005; Honnold et al., 2005) and manatees (Dubey et al., 2003; Buergelt and Bonde, 1983). Cerebral toxoplasmosis and sarcocystosis have been identified as significant causes of mortality in a southern sea otter, *Enhydra lutris nereis* (Cole et al., 2000; Kreuder et al., 2003). Therefore, it seems to be of paramount importance to determine the distribution and effect of this incidental, but pathogenic parasite of harbor porpoises from the threatened Baltic Sea population. So far such research has not been conducted here; this would require a change in the methodological approach and an expansion of the spectrum of the methods used.

Undoubtedly, the main research elements were analyzes of species diversity of parasitofauna and the functioning of individual parasite-host systems, including the relationship with the host (specificity, topical and topographic preferences), as well as that of the level of infection for a given species and the impact on the host. Another important issue addressed by the present study is the total load placed on the host by the parasites. Many of the examined harbor porpoises were characterized by the co-occurrence of several species of parasites, some of which were found to inhabit the same, or similar habitats, e.g. the respiratory nematodes. In such cases, it is important to obtain physical observations or case studies, as these enable the analysis of parasites of a specific host individual. In the present study, the largest (probably the oldest) specimen no. 68, a female porpoise, had the largest parasite load: 3940 specimens from five species, located in various organs, with a tropism to the ears and gastrointestinal tract. Such infection intensity not only undoubtedly results in reduced fitness and adaptability to environmental conditions, but may have an impact on the overall health of the animal and its survival. Although the parasite communities should be analyzed not only in terms of quantity, but also in qualitative terms. Some parasites, as a result of the long-term evolution of the parasite-host system, are well adapted to function in a given host, well tolerated and usually non-pathogenic. However, in this context, parasites that are less specific or new to the host, obtained as a result of favorable conditions, e.g. environmental changes, can more dangerous (Izdebska et al., 2020).

Against the global checklist of the parasites of the harbor porpoise*,* including 55 taxa (46 helminths), the list of parasites for the nominative subspecies from the Baltic Sea subpopulation is rather limited, being only 10 taxa. However, it should be taken into account that many records from other subspecies or populations in other regions are only singular or incidental. The parasitofauna may be influenced by various environmental factors, including the presence and availability of intermediate and paratenic hosts or other final hosts; these can serve as a reservoir of parasites typical of the harbor porpoise or as a potential source of infection with sporadic or incidental parasites. The species diversity of parasites in the harbor porpoises from the Baltic subpopulation appears small, even compared to neighboring subpopulations of this subspecies. However, it is important to note that only a relatively small number of hosts were examined in this study. The small size of this critically-endangered population is undoubtedly a limitation when conducting this type of survey, and the small number of specimens examined allows the detection of only the most common parasite species; even so, it should be emphasized that the prevalence is undoubtedly very high, reaching 87%, with a mean infection intensity of approx. 724 specimens.

It is difficult to compare this data to other research, as the body of evidence on harbor porpoises from this subpopulation is derived from just five publications. Of these, three examine only a single species in various contexts (Łukasiak, 1939; Szefer et al., 1998; Kijewska et al., 2003), one is based on data from different subpopulations in the context of the harbor porpoise health (Siebert et al., 2020), while the other, directly preceding the present study, recorded five helminth species in 17 harbor porpoises from the Polish zone of the Baltic Sea, with a comparable or lower prevalence of infection (5.9–88.2%, 9.0–163 ind.) (Rokicki et al., 1997).

Although the research carried out so far cannot unequivocally indicate that the level of Baltic harbor porpoise parasitic infection is increasing, it nevertheless demonstrates the constant presence of a parasite population with high importance for these mammals, especially respiratory nematodes. The parasites are widely dispersed with this porpoise population and although their presence in the host is not tantamount to the development of parasitoses, infected individuals are undoubtedly a significant reservoir of parasites. In turn, the increasing pace of change in environmental conditions, especially those related to human pressure, local and global climate change, may adversely affect the fitness or the level of immunity of marine mammals, reflected in increasing susceptibility to the development of diseases. It is worth noting that the current level of intensity of infection, and thus the parameter directly illustrating the influence of parasites on the hosts, is very high. This doubtlessly impairs the fitness, adaptation capacity or even health status of the host, affecting survival and reproduction. It should be remembered that the functioning of the Baltic Sea ecosystem, all its elements including porpoises, is influenced by various factors directly or indirectly related to human activity (global climate changes, pollution, fisheries management, etc.). While some factors (various types of pollution and contamination) are limited by protective measures, others are intensified (Rheinheimer, 1998; Elmgren, 2001; Garnaga, 2012). Thus, the ecosystem of the Baltic Sea is a dynamic system, to which organisms living here must adapt. Perhaps the factors resulting in the decline in the number of fish populations (eutrophication, oxygen deficiency, overfishing) (Jonzén et al., 2002), with the simultaneous appearance of invasive species (e.g. gobies) here (Sapota and Skóra, 2005; Schrandt et al., 2016), are important for the formation of the porpoise diet. Which, in turn, is important in the context of the pathways of infection and spread of parasites, or the condition and well-being of these mammals.

Certainly, the individual and random studies did not reflect all aspects of the occurrence and impact of parasitofauna on the fitness and the health status of harbor porpoises from the Baltic Proper subpopulation. In spite of the fact that this subpopulation is the most endangered of all the porpoise populations, the body of research is scarce and requires further supplementation. The presence and level of infection of these harbor porpoises requires ongoing monitoring can provide an important insight into not only the current status of the population, but also the changes to which it is subject.

## Declaration of competing interest

Authors have no conflict of interest to declare.
